# Diagnostic performance of patient self‐collected oral swab (tongue and cheek) in comparison with healthcare worker‐collected nasopharyngeal swab for severe acute respiratory syndrome coronavirus‐2 detection

**DOI:** 10.1111/apm.13266

**Published:** 2022-09-08

**Authors:** Arati Mane, Shilpa Jain, Ankita Jain, Vijay Nema, Swarali Kurle, Vandana Saxena, Michael Pereira, Atul Sirsat, Gaurav Pathak, Vikalp Bhoi, Shailaja Bhavsar, Samiran Panda

**Affiliations:** ^1^ ICMR‐National AIDS Research Institute Pune India; ^2^ Old Bhosari Hospital Pune India; ^3^ Division of Epidemiology and Communicable Diseases Indian Council of Medical Research New Delhi India

**Keywords:** SARS‐CoV‐2, oral swab, nasopharyngeal swab, RT‐PCR

## Abstract

The present study was conducted to compare the performance of patient self‐collected oral swab (OS) with healthcare worker (HCW)‐collected nasopharyngeal swab (NPS) for SARS‐CoV‐2 detection by reverse transcription polymerase chain reaction (RT‐PCR) in real‐world setting. Paired OS and NPS were collected from 485 consecutive individuals presenting with symptoms of coronavirus disease‐19 (COVID‐19) or asymptomatic contacts of COVID‐19 cases. Both specimens were processed for RT‐PCR and cycle threshold (Ct) value for each test was obtained. Positive percent agreement (PPA), negative percent agreement (NPA), overall percent agreement (OPA) and kappa were calculated for OS RT‐PCR compared with NPS RT‐PCR as reference. A total of 116/485 (23.9%) participants were positive by NPS RT‐PCR. OS had PPA of 71.6%, NPA of 98.8%, OPA of 92.4% and kappa of 0.771. Almost all participants (483/485, 99.6%) reported OS as a convenient and comfortable sample for SARS‐CoV‐2 testing over NPS. All participants with Ct values <25 and majority (90.8%) with Ct values <30 were detected by OS. To conclude, OS self‐sampling was preferred in comparison with NPS due the ease and comfort during collection. The performance of OS RT‐PCR for SARS‐CoV‐2 detection, however, was sub‐optimal in comparison with NPS RT‐PCR.

Severe acute respiratory syndrome coronavirus 2 (SARS‐CoV‐2), the causative agent of coronavirus disease‐19 (COVID‐19) wreaked havoc across the globe. The crucial strategy for controlling transmission relies on timely detection and isolation of COVID‐19 infected individuals. The most widely used diagnostic test for the detection of SARS‐CoV‐2 is reverse transcription polymerase chain reaction (RT‐PCR) with nasopharyngeal swab (NPS) specimen (1, 2). However, NPS collection requires travel to a designated collection facility, availability of trained healthcare workers (HCW) and leads to aerosol generation through inducement of coughing or sneezing with potential risk of viral transmission to HCW (3, 4). NPS collection also causes pain and discomfort to individuals and may be associated with severe complications at times (5). Moreover, it poses difficulties in pediatric and geriatric population as they are often non‐cooperative, in individuals with nasal anomalies and psychiatric disorders, and in cases where frequent testing is required (6, 7).

Saliva has been demonstrated as an easy, non‐invasive option for detection of SARS‐CoV‐2 having high concordance with NPS (8, 9, 10). However, there are challenges associated with saliva collection and processing, which has prompted the evaluation of oral swabs as an alternative specimen for SARS‐CoV‐2 detection (11, 12). Oral swab collection is easy and requires no special arrangement for privacy or aerosol control (13). A few studies have evaluated their utility for SARS‐CoV‐2 detection but with conflicting results. Most of these studies have been conducted on small sample sizes and have included known SARS‐CoV‐2‐positive patients with unspecified information on symptomatic or asymptomatic status (14, 15, 16, 17).

The present study was conducted against this backdrop to evaluate the diagnostic performance of patient self‐collected oral swab (OS) specimen (tongue and inside of cheek surfaces) for SARS‐CoV‐2 detection by RT‐PCR in comparison with HCW collected NPS specimen.

## METHODS

### Study participants and specimens

Participants for the study were enrolled from among consecutive individuals attending the COVID‐19 facility at Old Bhosari Hospital (OBH), Pune, and presenting with symptoms of COVID‐19 infection and those who were asymptomatic high‐risk contacts of COVID‐19 cases. Written informed consent was obtained from all participants prior to their enrollment in the study. Participants were excluded if they were less than 18 years of age and either of the NPS or OS specimen could not be collected. Information on socio‐demographic profile, clinical symptoms, COVID‐19 vaccination status and views regarding preference for sample collection and comfort were collected.

Participants were provided pictorial illustration of the procedure for OS collection. Boards illustrating the procedure for OS collection were displayed at different sites in the waiting area of the COVID‐19 facility. Paired NPS (collected by HCWs) and self‐administered OS specimens were obtained using flocked nylon swabs. OS was collected by the participants by rubbing the tip of the swab on the dorsal and ventral surface of the tongue and the inside of each cheek surface for 10 s each. The swab was put in viral transport medium (VTM) (HiViral Transport Medium, HiMedia Laboratories Pvt. Limited, Maharashtra, India). This was followed by collection of NPS by HCW. NPS and OS specimens in VTM were transported to ICMR‐NARI COVID‐19 laboratory for RT‐PCR testing (18). The study was conducted during April to June 2021. This was the period when the second COVID‐19 wave was present in India and the predominant circulating variant was B.1.617.2 (Delta). Other SARS‐CoV‐2 variants including, B.1.1.7 (Alpha), B.1.351 (Beta) and B.1.1.28.1 (Gamma), B.1.1.28.2 (Zeta), B.1.617.1 68 (Kappa) and B.1.617.3 were also present in varying proportions (19).

### RT‐PCR assay

Both OS and NPS were tested for SARS‐CoV‐2 RT‐PCR. Viral RNA was isolated from the VTM using the MDS Viral RNA Extraction kit (MetaDesign Solutions, Gurgaon, India) and tested for SARS‐CoV‐2 with the Covidsure Multiplex RT‐PCR kit (Trivitron Healthcare Labsystems Diagnostics, Chennai, India) on the CFX96 Real‐Time Detection System (Bio‐Rad, Hercules, CA, USA). The kit targets the *E* and ORF genes of SARS‐CoV‐2 and uses RPP30 human gene as internal control. Result was considered positive if cycle threshold (Ct) value was less than 35 for the genes tested. The ORF gene was considered for analysis of Ct values.

### Statistical analysis

Descriptive statistics are presented as means for continuous and as proportions for categorical variables. Independent *t*‐test or Mann–Whitney test was applied to continuous variables and Fisher's exact test to the categorical variables for comparisons. The results of OS RT‐PCR were compared to NPS RT‐PCR and positive percent agreement (PPA), negative percent agreement (NPA), overall percent agreement (OPA) and kappa (k) with 95% confidence interval (95% CI) were calculated. Pearson correlation analysis was used to evaluate Ct values between the NPS and OS specimens. GraphPad statistical software (GraphPad Software Inc, San Diego, CA, USA) and online MedCalc statistical calculators (https://www.medcalc.org/calc/) were used for statistical analyses. p value less than 0.05 was considered to be of statistical significance.

## RESULTS

### Participant characteristics

A total of 485 participants were enrolled in the study. The demographic characteristics of these participants are presented in Table 1. The mean time from the onset of symptoms to sample collection was (mean ± standard deviation) 2.6 ± 1.4 days. The presenting symptoms were dry cough (74.2%), fever (57.9%), aches/tiredness (57.3%), sore throat (51.7%), conjunctivitis (15.2%), and diarrhea (8.4%).

**Table 1:** Characteristics of the study participants

Characteristic	Total (n = 485)	SARS‐CoV‐2‐positive (n = 116)	SARS‐CoV‐2‐negative (n = 369)	p
Age (years) mean (±SD)	30.3 ± 10.8	33.5 ± 12.2	29.3 ± 10.2	0.0003
Sex				
Male	360 (74.2%)	79 (68.1%)	281 (76.2%)	0.08
Female	125 (25.8%)	37 (31.9%)	88 (23.8%)	
Symptomatic				
Yes	178 (36.7%)	80 (69%)	98 (26.6%)	<0.00001
No	307 (63.3%)	36 (31%)	271 (73.4%)	
Duration since symptom onset	2.6 ± 1.4	2.7 ± 1.4	2.5 ± 1.3	0.156
COVID‐19 vaccine taken				
Yes	76 (15.7%)	19 (16.4%)	57 (15.4%)	0.883
No	409 84.3%)	97 (83.6%)	312 (84.6%)	

### Performance of OS RT‐PCR as compared to NPS RT‐PCR

A total of 970 samples (485 NPS and 485 OS) were tested for SARS‐CoV‐2 by RT‐PCR. One hundred and sixteen (23.9%) participants were positive by NPS RT‐PCR. SARS‐CoV‐2‐positive individuals were older and more likely to be symptomatic as compared to the SARS‐CoV‐2‐negative participants (Table 1).

The diagnostic performance of OS specimen is presented in Table 2. Overall, OS had PPA of 71.6% (95% CI = 62.6–79), NPA of 98.8% (97.2–99.6), *OPA* of 92.4% (89.7–94.4), and k = 0.771 (95% CI = 0.701–0.840) as compared to NPS,

**Table 2:** Comparison of diagnostic performance of Oral swab RT‐PCR

Oral swab RT‐PCR	Nasopharyngeal swab RT‐PCR			
		Positive	Negative	
All participants	Positive	83	4	PPA: 71.6% NPA: 98.9%
	Negative	33	365	
Symptomatic individuals	Positive	68	0	PPA: 85% NPA: 100%
	Negative	12	98	
Asymptomatic individuals	Positive	15	4	PPA: 41.7% NPA: 98.5%
	Negative	21	267	

NPA, negative percent agreement; PPA, positive percent agreement.

Among symptomatic individuals, OS had PPA of 85% (75.6–91.2), NPA of 100% (96.2–100), *OPA* of 93.3% (88.6–96.1) and k = 0.862 (0.787–0.937), while among asymptomatic individuals, OS had PPA of 41.7% (27.1–57.8), NPA of 98.5% (96.3–99.4), OPA of 91.9% (88.3–94.4), and k = 0.505 (0.340–0.671).

Almost all participants (483/485, 99.6%) expressed preference for OS as the most convenient and comfortable sample collection approach for SARS‐CoV‐2 testing as compared to NPS.

### Comparison of Ct values (proxy for viral load) in OS and NPS

**Fig. 1:**
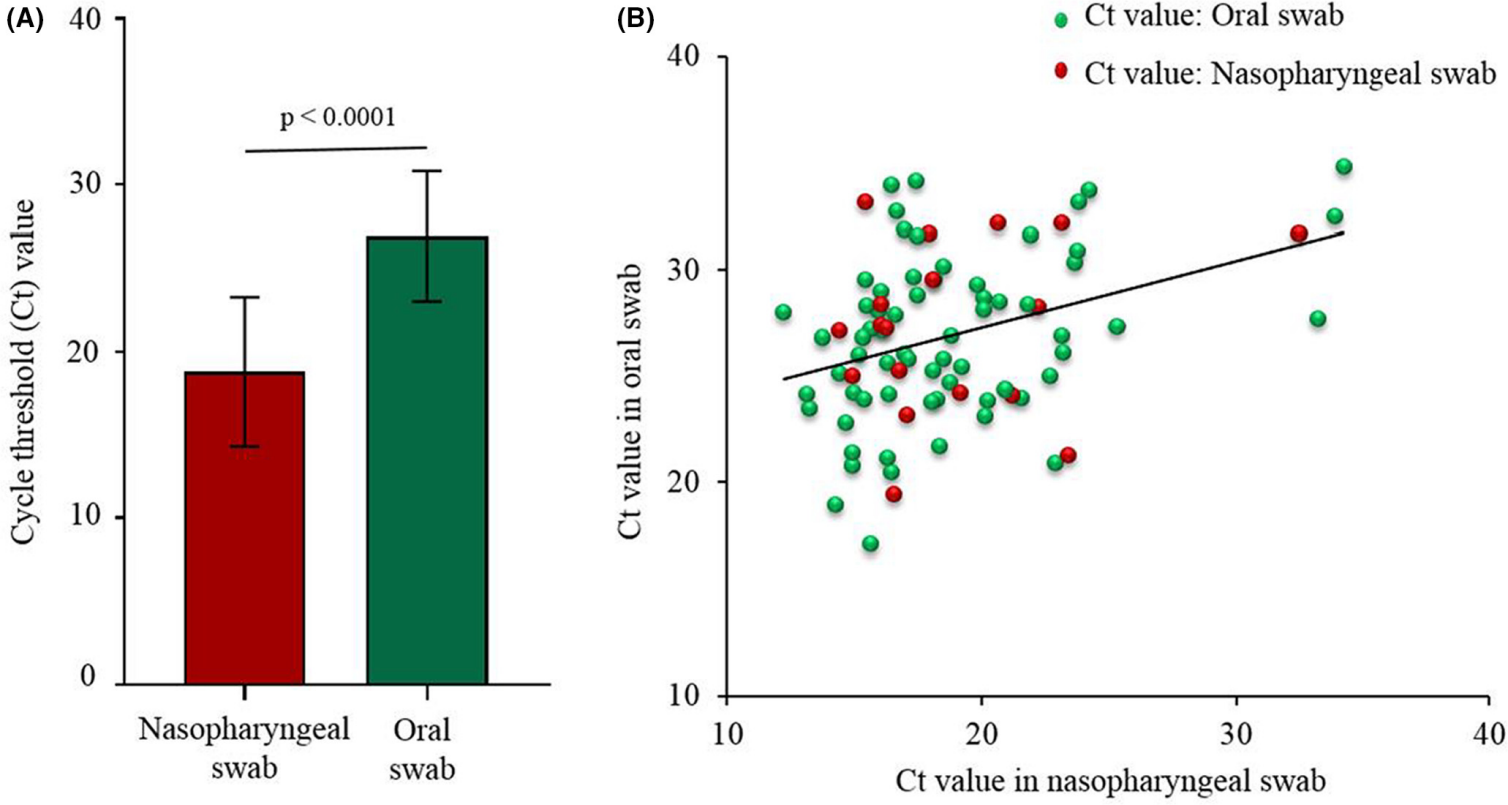
Cycle threshold (Ct) values in nasopharyngeal and oral swab specimens. (A) Mean cycle threshold values in nasopharyngeal and oral swab specimens. (B) Association between cycle threshold (Ct) values of nasopharyngeal and oral swabs.

The mean (± standard deviation) Ct values in NPS versus OS were 18.8 (± 4.5) and 26.9 (± 3.9), respectively, p < 0.0001 (Fig. 1A). A weak positive correlation was observed between the Ct values of NPS and oral swab, r = 0.356, p < 0.001 (Fig. 1B).

**Fig. 2:**
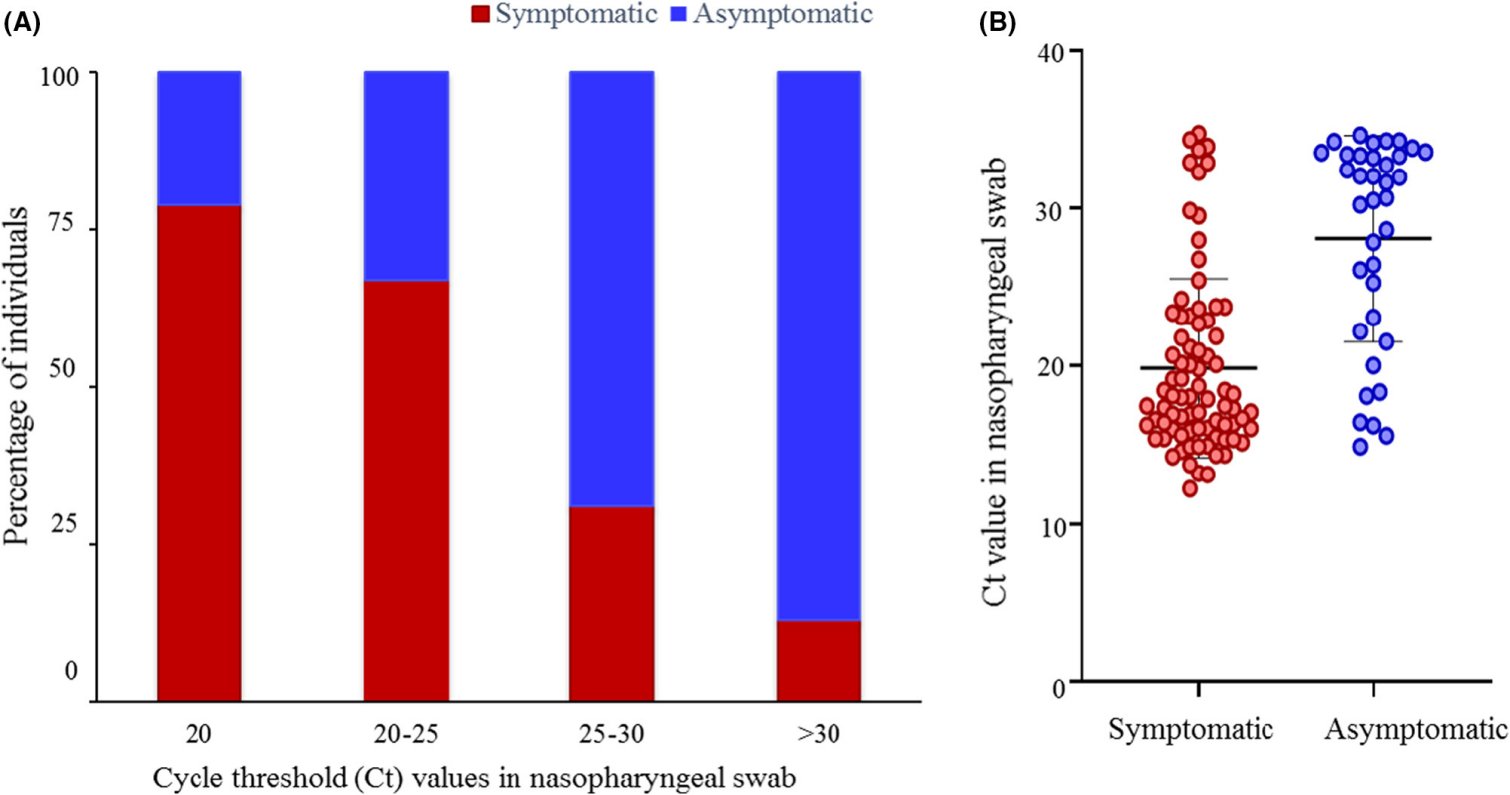
Cycle threshold (Ct) values among symptomatic and asymptomatic individuals. (A) Distribution of samples by cycle threshold values among symptomatic and asymptomatic individuals. (B) Mean cycle threshold values among symptomatic and asymptomatic individuals.

Figure 2A depicts the distribution of samples by Ct values among symptomatic and asymptomatic individuals. Significantly greater number of samples from asymptomatic individuals (58.3%) had Ct values more than 30 as compared to symptomatic individuals (10%), p < 0.0001. All samples (78/78, 100%) with Ct values less than 25 and majority of samples (79/87, 90.8%) with Ct values less than 30 from both symptomatic and asymptomatic individuals were positive by OS. The mean Ct values in symptomatic versus asymptomatic individuals were 19.9 ± 5.7 and 28.1 ± 6.5, respectively, p < 0.0001 (Fig. 2B).

**Fig. 3:**
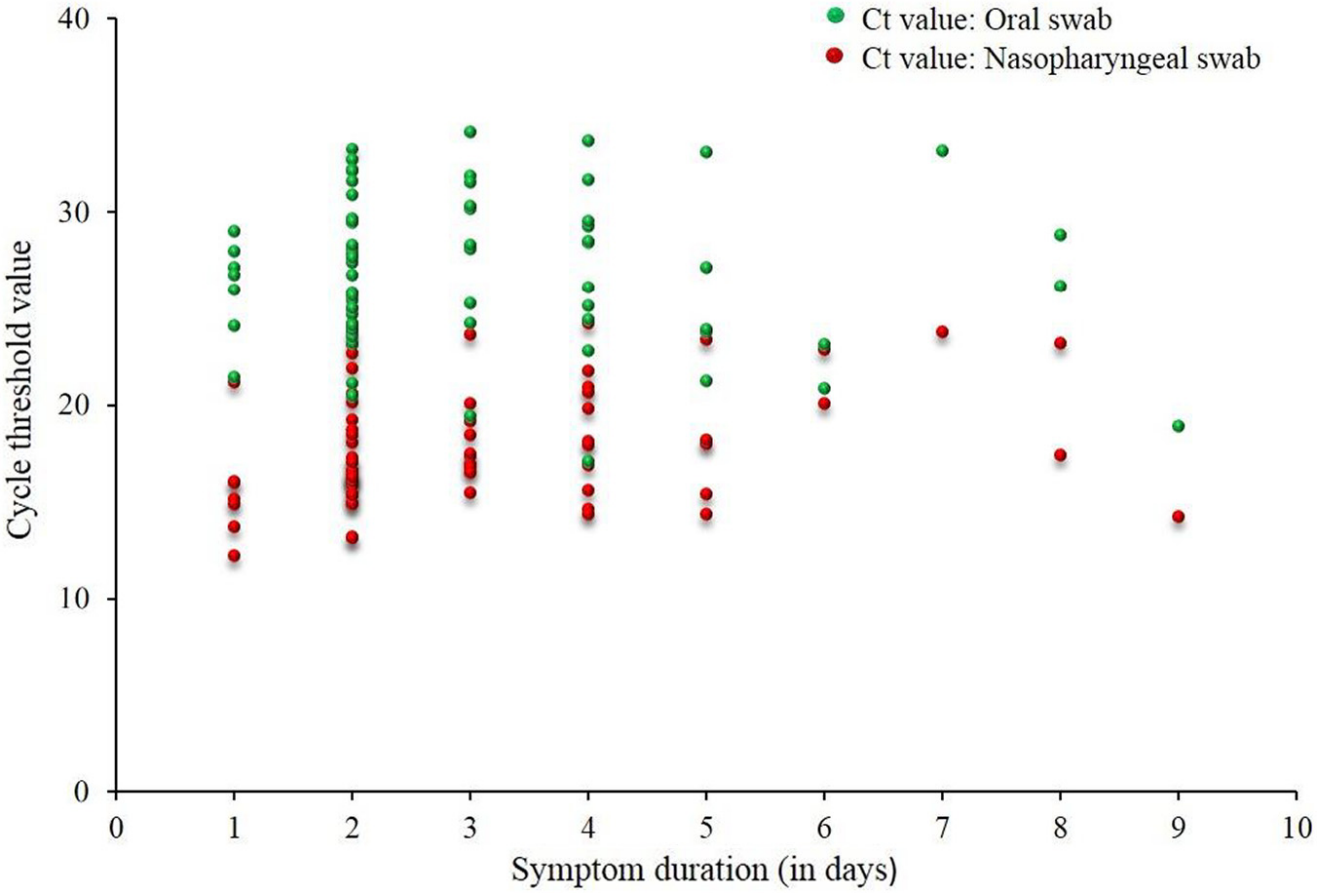
Association between cycle threshold (Ct) values and duration of symptoms.

No significant association between duration of symptoms and Ct values was observed, r = 0.12, p = 0.126 (Fig. 3).

## DISCUSSION

The current diagnostic standard for SARS‐CoV‐2 detection involves testing of NPS specimen, a relatively invasive one, with RT‐PCR. NPS collection is not suitable, particularly, in pediatric and geriatric age groups, in individuals with certain medical conditions and for frequent testing. Thus, search for non‐invasive alternative to NPS is ongoing. Anterior nasal cavity and mid‐turbinate sampling are being reported as substitutions to NPS (20, 21).

The oral cavity has been a region of particular interest for SARS‐CoV‐2, owing to the high expression of angiotensin converting enzyme‐2 (ACE‐2) receptors in different parts such as the dorsum of tongue, gingiva, and buccal mucosa (22, 23). These parts serve as entry points for the virus and facilitate viral shedding, justifying the exploration of OS for SARS‐CoV‐2 detection. OS has been previously applied for molecular detection of microorganisms like *Mycobacterium tuberculosis* (24, 25, 26), *Human papillomavirus* (27, 28), *Ebolavirus* (29), and *Leishmania donovani* (30), demonstrating its expanding potential for molecular detection of infectious etiologies and the need for evaluation of this alternative specimen for SARS‐CoV‐2 detection.

Prior studies have evaluated oral swab specimen for SARS‐CoV‐2 detection and reported sensitivities ranging from 56.7% to 89.8% (14, 15, 16, 17). The wide variations in the diagnostic performance across studies could be attributed to the population groups studied, the collection procedure and the timing of specimen collection and testing.

In the present study, OS showed good NPA for SARS‐CoV‐2 detection among both symptomatic (100%) and asymptomatic (98.5%) individuals. The PPA, however, was 85% among symptomatic and 41.7% among asymptomatic individuals. It is important to note here that the viral load in the specimen plays an important role in the diagnostic accuracy of the test. The lower PPA observed in the present study can be attributed to the lower viral load (as indicated by Ct values) detected in the OS specimen. This difference may be due factors related to specimen collection and processing as well as to the pathophysiological basis that nasopharyngeal ciliated epithelial cells act as a viral reservoir to a greater degree as compared to oral squamous epithelial cells (31, 32). Similar observation has been reported with the use of anterior nasal swab for SARS‐CoV‐2 detection, where the lower sensitivity was attributed to rapid reduction of viral loads in the anterior nares than in the nasopharynx with disease progression (20).

OS detected all specimens with Ct values less than 25 and 90.8% specimens with Ct value less than 30. Thus, most individuals capable of disease transmission were picked up with this sampling method. The higher Ct values in majority of specimens from asymptomatic individuals explains the lower PPA observed in them as compared to symptomatic individuals. OS detected four additional individuals who tested negative by NPS RT‐PCR, supporting the observation that testing of a single anatomic site may miss a few SARS‐CoV‐2 cases (11, 14).

NPS will remain the gold standard specimen for SARS‐CoV‐2 diagnostic screening. We further acknowledge that sensitivity similar to what was observed among symptomatic individuals with OS may be attained using a validated rapid antigen test with NPS and be a cost‐effective option. However, oral swabs are amenable to self‐collection, speed specimen collection and precludes the need of a trained HCW. OS may have utility for use in resource‐limited settings where facility for NPS collection is unavailable and in situations where frequent testing is required.

Our study had strengths. It was conducted in a busy urban COVID‐19 facility in a real‐world setting. We recruited adequate number of both symptomatic and asymptomatic individuals that allowed us to examine the performance of OS in varying clinical situation and in order to maintain homogeneity in assessment, samples were collected at clearly defined time‐points. The current study demonstrated the feasibility of self‐collection of OS by participants. The limitations of this study were that it was conducted in a single center and we did not include pediatric population and individuals with medical conditions not suitable for NPS collection.

In conclusion, OS self‐sampling was preferred in comparison with NPS due the ease and comfort during collection as demonstrated in our study. The performance of OS RT‐PCR for SARS‐CoV‐2 detection, however, was sub‐optimal in comparison with NPS RT‐PCR.

###  

We thank the staff at OBH for their support in participant enrollment and the COVID‐19 diagnostic team at ICMR‐NARI for routine RT‐PCR.

## CONFLICT OF INTEREST

The authors declare that there was no conflict of interest in the conduct of this study.

## FUNDING INFORMATION

The work was carried out through ICMR‐NARI intramural support.
